# HbBIN2 Functions in Plant Cold Stress Resistance through Modulation of HbICE1 Transcriptional Activity and ROS Homeostasis in *Hevea brasiliensis*

**DOI:** 10.3390/ijms242115778

**Published:** 2023-10-30

**Authors:** Yi-Min Qiu, Jing Guo, Wei-Zeng Jiang, Jia-Hui Ding, Ru-Feng Song, Jian-Long Zhang, Xi Huang, Hong-Mei Yuan

**Affiliations:** School of Breeding and Multiplication (Sanya Institute of Breeding and Multiplication), School of Tropical Agriculture and Forestry, Hainan University, Sanya 572025, China; hy0206070@hainmc.edu.cn (Y.-M.Q.); guojing2089@163.com (J.G.); 21210901000020@hainanu.edu.cn (W.-Z.J.); 22220951310110@hainanu.edu.cn (J.-H.D.); songrufeng2021@163.com (R.-F.S.); 22210901000062@hainanu.edu.cn (J.-L.Z.)

**Keywords:** abiotic stresses, *Hevea brasiliensis*, GSK3-like kinase, ROS, transcriptional regulation

## Abstract

Cold stress poses significant limitations on the growth, latex yield, and ecological distribution of rubber trees (*Hevea brasiliensis*). The GSK3-like kinase plays a significant role in helping plants adapt to different biotic and abiotic stresses. However, the functions of GSK3-like kinase BR-INSENSITIVE 2 (BIN2) in *Hevea brasiliensis* remain elusive. Here, we identified *HbBIN2s* of *Hevea brasiliensis* and deciphered their roles in cold stress resistance. The transcript levels of *HbBIN2s* are upregulated by cold stress. In addition, HbBIN2s are present in both the nucleus and cytoplasm and have the ability to interact with the INDUCER OF CBF EXPRESSION1(HbICE1) transcription factor, a central component in cold signaling. *HbBIN2* overexpression in *Arabidopsis* displays decreased tolerance to chilling stress with a lower survival rate and proline content but a higher level of electrolyte leakage (EL) and malondialdehyde (MDA) than wild type under cold stress. Meanwhile, *HbBIN2* transgenic *Arabidopsis* treated with cold stress exhibits a significant increase in the accumulation of reactive oxygen species (ROS) and a decrease in the activity of antioxidant enzymes. Further investigation reveals that HbBIN2 inhibits the transcriptional activity of HbICE1, thereby attenuating the expression of *C-REPEAT BINDING FACTOR (HbCBF1).* Consistent with this, overexpression of *HbBIN2* represses the expression of CBF pathway cold-regulated genes under cold stress. In conclusion, our findings indicate that HbBIN2 functions as a suppressor of cold stress resistance by modulating HbICE1 transcriptional activity and ROS homeostasis.

## 1. Introduction

Cold stress is a major environmental stress that inhibits plant growth, restricts plant geographic distribution, and affects crop productivity and quality [1,2]. Therefore, the improvement of cold tolerance is an important goal in crop breeding. Being sessile, plants have evolved a series of adaptation mechanisms to withstand adverse growth environments [3,4]. Extensive studies have been conducted regarding the specific mechanisms of plant response to low temperature in model plants, and significant progress has been made in analyzing and recognizing complex signaling pathways. These pathways include cold sensors, secondary messengers, protein kinases, transcription factors, and their target genes [5,6,7,8]. Under cold stress, COLD1 (CHILLINGTOLERANCE DIVERGENCE 1) perceives low-temperature stress and triggers Ca^2+^ signaling, leading to downstream gene responses, among which CBF transcription factors and their direct target genes activate transcriptional regulatory cascades [9]. The ICE-CBF-COR transcriptional cascade is critical in plant reactions to exposure to cold. Cold stress rapidly stimulates CBF expression in this pathway, upregulating several COR (cold-regulated) genes to improve plant cold resistance [2,10,11]. ICE1 (Inducer of CBF expression1) is the first identified transcription factor that positively regulates CBF3 to enhance plant cold tolerance [12]. Besides ICEs, the transcriptions of CBFs are also regulated by CAMTA3 (calmodulin-binding transcription activator 3), EIN3, MYB, CCA19 (circadian clock associated1), PIFs (phytochrome-interacting factors), BTF3s, and 14-3-3 proteins [13,14,15,16,17].

Brassinosteroids (BRs), a class of steroid plant hormones, have crucial functions in plant growth and development by promoting cell elongation, differentiation, and division [18,19]. Moreover, BRs have been recognized for their role in enhancing plant tolerance to diverse abiotic stresses, including salt, drought, heat, and cold stresses [20,21,22]. In the presence of BR, the BR binds to its receptor BRASSINOSTEROID insensitive1(BRI1), activating BRI1 kinase activity by changing its conformation [23,24]. The activated BRI1 kinase then phosphorylates and activates BRI1-associated kinase 1 (BAK1) co-receptor, initiating a phosphorylation cascade that inhibits BRASSINOSTEROID-INSENSITIVE2 (BIN2), activating BR-dependent genes transcription [25,26]. BIN2, a glycogen synthase kinase 3 (GSK3), is a key negative regulator of the BR signaling pathway and controls how plants respond to both abiotic and biotic stressors [27,28,29]. In the context of rice, OsqGL3 (quantitative trait locus regulating grain length 3) has been identified as a negative regulator of brassinosteroid signaling. This is achieved by modulating the phosphorylation level and stability of OsGSK3, thereby regulating the phosphorylation of OsBZR1 (Oryza sativa BRASSINAZOLE RESISTANT1) [30]. In cotton, GhBIN2 phosphorylates and destabilizes GhJAZ proteins to negatively regulate plant defense against *Verticillium dahlia* [31]. The GSK3-like kinase AtBIN2 in *Arabidopsis* functions as a molecular switch to precisely regulate plant response to salt stress and plant growth by cooperating with the calcium sensors SOS3 and SCaBP8 [32]. Additionally, it has been reported that *AtBIN2* in *Arabidopsis* promotes the phosphorylation and degradation of ICE1, thus decreasing plant cold tolerance [28]. The functional studies of important genes in the BR pathway in *Hevea brasiliensis* are very limited. Recently, five *HbBRI1s* and four *HbBAK1s* have been identified [33]. The expression of *HbBIN2* was found to be significantly induced after treatment with ethephon [34]; however, the functions of the *Hevea brasiliensis BIN2* genes in cold stress remain elusive.

Natural rubber is a vital industrial raw resource that is utilized extensively in transportation, medicinal and health treatment, national defense, and military applications. Due to its excellent comprehensive performance, natural rubber cannot be completely replaced by synthetic rubber so far [35]. The only economic source of natural rubber is the rubber tree (*Hevea brasiliensis*); however, it is susceptible to low temperatures [36]. Cold stress can cause damage to the rubber tree, leading to a reduced yield of rubber [37,38]. Therefore, identifying potential genes involved in the low-temperature response of rubber trees and elucidating the mechanisms that regulate cold tolerance will help develop strategies to breed cold-resistant rubber tree varieties. Despite extensive studies on the response mechanisms of plants to cold stress in model plants, the understanding of the molecular mechanisms behind rubber plants’ response to low temperature is still limited. This is because of the delayed completion of the genome sequencing and immature transgenic technology of rubber trees. The essential genes *HbCBF1* and *HbICE1/HbICE2* of the ICE-CBF signaling pathway in rubber trees were recently identified, and their favorable functions in cold tolerance in *Hevea brasiliensis* were investigated [36,38,39]. However, the pivotal regulators of the ICE-CBF signaling pathway in *Hevea brasiliensis* still need to be further studied. In the present study, *BIN2s (HbBIN2s)* of *Hevea brasiliensis* were identified, and their roles in response to cold stress were also elucidated. HbBIN2–1 and HbBIN2–3 were found to physically interact with HbICE1, which is a key transcription factor of the ICE-CBF signaling pathway in *Hevea brasiliensis* that positively regulates plant freezing tolerance. In addition, HbBIN2–1 inhibits the transcriptional activity of HbICE1, thus reducing the expression of *HbCBF1*, the direct target of transcription factor HbICE1. Consistently, overexpression of *HbBIN2–1* or *HbBIN2–3* suppressed the freezing tolerance with increased electrolyte leakage, MDA content, and ROS accumulation under cold stress. These findings suggest that *HbBIN2–1* or *HbBIN2–3* has a negative impact on low-temperature stress by modulating HbICE1′s transcriptional activity and ROS homeostasis.

## 2. Results

### 2.1. Isolation and Bioinformatics Analysis of HbBIN2s

**Figure 1:**
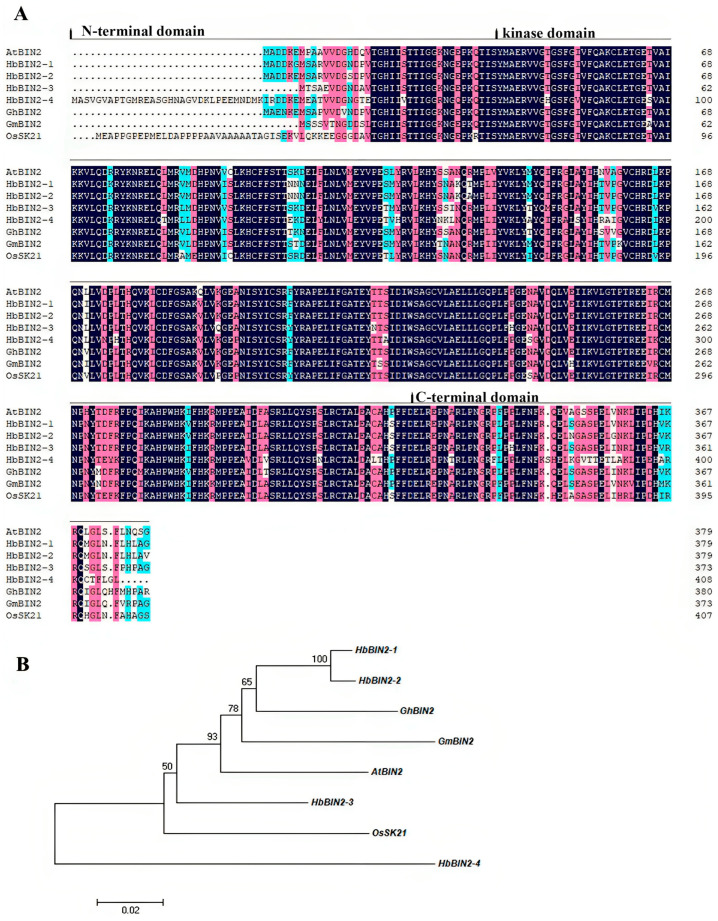
Sequence analyses of HbBIN2s. (**A**) Amino acid sequence alignment of HbBIN2s and BIN2 proteins from other plants. Identical and similar residues are shown in blue and pink backgrounds, respectively. The N-terminal domain, kinase domain, and C-terminal domain are labeled. (**B**) Phylogenetic tree depicting the relationship of HbBIN2s proteins between *Hevea brasiliensis* and other plant BIN2 proteins. Species name abbreviations are as follows: At, *Arabidopsis*; Hb, *Hevea brasiliensis*; Gh, *Gossypium hirsutum*; Gm, *Glycine max*; and Os, *Oryza sativa*.

Using the *A. thaliana* HbBIN2 protein sequence as a query sequence, NCBI online BLAST analysis discovered four *HbBIN2s* from *Hevea brasiliensis*. These four *HbBIN2* genes were named *HbBIN2–1*(accession no. OR394642), *HbBIN2–2* (accession no. OR394643), *HbBIN2–3* (accession no. OR394644), and *HbBIN2–4* (accession no. OR394645) according to their homology with *A. thaliana AtBIN2*. The isolated *HbBIN2s* contained 1143-bp, 1143-bp, 1125-bp, and 1227-bp open reading frames, respectively. HbBIN2s have strong sequence similarity to BIN2s proteins from other plants according to multiple amino acid sequence alignment. All members of HbBIN2s possessed a C-terminal domain, a variable N-terminal region, and a highly conserved domain of 285 amino acids, which represents a serine/threonine protein kinase catalytic domain (Figure 1A). Based on the full amino acid sequences of HbBIN2s and other plant BIN2 proteins, a phylogenetic tree was created, in which both HbBIN2–1 and HbBIN2–2 were most closely related to BIN2 from *Gossypium hirsutum* (Figure 1B).

### 2.2. Expression Patterns of HbBIN2s

**Figure 2:**
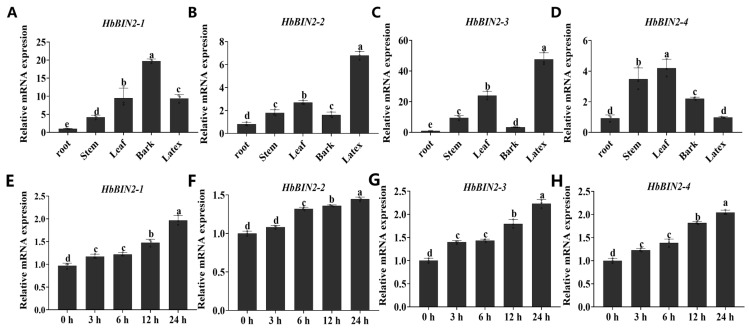
Expression patterns of the members of the HbBIN2 gene family. (**A**–**D**) qRT-PCR analysis of four *HbBIN2* expression levels in different tissues, including roots, leaves, latex, bark, and stems. *HbeIF2* was used as the reference gene. (**E**–**H**) Expression analysis of *HbBIN2s* genes in leaves of 6-month-old *Hevea brasiliensis* treated at 10 °C for the indicated time. Error bars represent the standard deviation based on three biological replicates. Different letters above the bars indicate significant differences at *p* < 0.05 (Duncan’s range test).

To understand the potential function of *HbBIN2s*, the tissue-specific expression pattern of *HbBIN2s* was investigated by qRT-PCR. Our findings revealed that *HbBIN2s* were universally expressed in all five tissues examined (roots, leaves, latex, bark, and stems). Notably, *HbBIN2–1* shows the highest expression of the bark among all tissues tested. The expression levels of *HbBIN2–2* and *HbBIN2–3* in latex were relatively higher than those in other tissues, and *HbBIN2–4* was expressed in the highest amount in the leaves. These data indicate that *HbBIN2s* highly expressed in specific tissues may play specialized roles in the corresponding tissues (Figure 2A–D). To explore the potential involvement of *HbBIN2s* in the response of *Hevea brasiliensis* to low temperatures, we conducted an examination of *HbBIN2s* expression under cold stress. Transcript levels of all four *HbBIN2* genes increased progressively with the cold treatment period (Figure 2E–H), suggesting that *HbBIN2* genes were involved in *Hevea brasiliensis* cold stress response.

### 2.3. Subcellular Location of HbBIN2s

**Figure 3:**
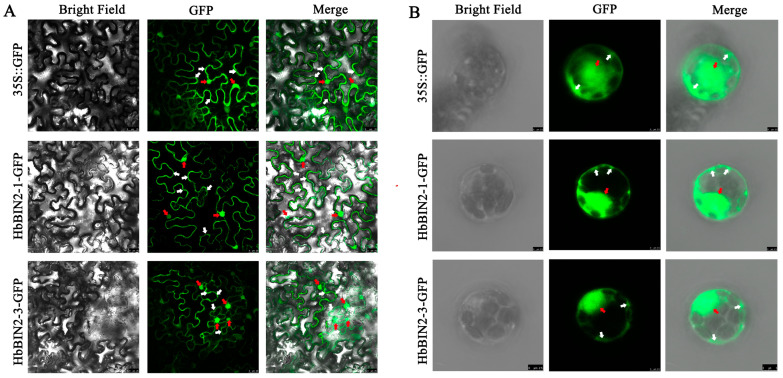
Subcellular localization of HbBIN2 proteins. GFP control vector or 35S::HbBIN2-GFP recombinant plasmid were transformed into tobacco (*Nicotiana benthamiana*) leaves (**A**) and protoplasts of *Hevea brasiliensis* (**B**). Representative images are shown for tobacco epidermal cells and *Hevea brasiliensis* protoplasts taken under GFP fluorescence and bright field. HbBIN2s in the cytoplasm and nucleus were indicated by white arrows and red arrows, respectively. The merged images are overlapped with the two images on the left. Bars = 25 μm.

All four HbBIN2 proteins in *Hevea brasiliensis* share considerably high sequence similarity with the AtBIN2 protein in *Arabidopsis,* with HbBIN2–1, HbBIN2–2, and HbBIN2–3 showing higher similarity with AtBIN2 protein than HbBIN2–4; thus, HbBIN2–4 was not selected for further study. Given that HbBIN2–1 and HbBIN2–2 have 98% amino acid sequence identity and may have very similar functions, we chose one of them, HbBIN2–1, for further study. Given their role as representative candidates, HbBIN2–1 and HbBIN2–3 were chosen as the key representatives for additional research. Emerging evidence has indicated that regulation of the subcellular localization of GSK3s significantly influences their biological functions in plants [29]. In *Arabidopsis*, SOS3/SCaBP8 alters BIN2 subcellular localization to regulate the salt stress response [32]. HSP90 regulates BIN2 kinase activity by modulating its subcellular localization [40]. However, the subcellular localization of BIN2 in *Hevea brasiliensis* remains unknown. To investigate the subcellular localization of HbBIN2, the HbBIN2–1 and HbBIN2–3 proteins were expressed in fusion with the GFP under the control of the CaMV 35S promoter. The fusion protein HbBIN2s-GFP and control GFP were transiently expressed in *N. benthamiana* leaf epidermal cells by infiltration with *Agrobacterium tumefaciens*. The microscopic examination revealed that the green fluorescence was dispersed across the entire cell harboring the control GFP vector, whereas the HbBIN2s-GFP fluorescence signal was primarily distributed in the cytoplasm and nucleus of the *N. benthamiana* cells with the 35S::HbBIN2s-GFP construct (Figure 3A). To further confirm the subcellular location of *HbBIN2s* in *Hevea brasiliensis,* the CDS of HbBIN2 was cloned into the transient expression vector PUC-35S-GFP and transformed into *Hevea brasiliensis* protoplasts. HbBIN2s were found in both the nucleus and the cytoplasm of *Hevea brasiliensis* protoplasts, which was consistent with the results obtained in *N. benthamiana* epidermal cells (Figure 3B). These results indicated that both HbBIN2–1 and HbBIN2–3 are localized in the nucleus and cytoplasm.

### 2.4. Overexpression of HbBIN2 Decreases Arabidopsis Cold Tolerance

**Figure 4:**
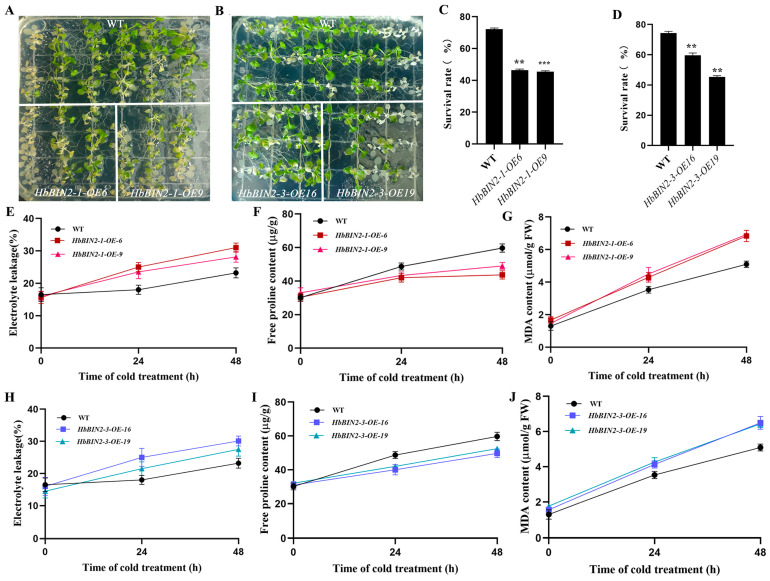
Overexpression of *HbBIN2* impairs cold tolerance in *Arabidopsis*. Freezing phenotypes of two-week-old *Arabidopsis* seedlings grown in 1/2 MS medium were pretreated at 4 °C for 3 days and then exposed to −20 °C for 2 h; the phenotype (**A**,**B**) and survival rates (**C**,**D**) were analyzed after the cold-treated seedlings were transferred to normal conditions for 3 days. Electrolyte leakage (**E**,**H**), proline content (**F**,**I**), and malondialdehyde (MDA) level (**G**,**J**) in the *35S::HbBIN2* transgenic and WT control plants before and after cold treatment. Data shown are mean ± SD of three independent experiments, and asterisks indicate significant differences at ** *p* < 0.01, *** *p* < 0.001 (Student’s *t*-test).

To validate the role of *HbBIN2s* in vivo, *Arabidopsis* plants overexpressing *HbBIN2* were generated. Two distinct transgenic lines containing high quantities of *HbBIN2–1* (OE-6 and OE-9) and *HbBIN2–3* (OE-16 and OE-19) transcripts were chosen for freezing tolerance studies. Under cold stress growth conditions, *HbBIN2–1/HbBIN2–3* overexpressing transgenic lines displayed substantially decreased tolerance to freezing stress in comparison with wild-type plants, and the survival rates of *HbBIN2–1/HbBIN2–3* transgenic lines were significantly lower than those of WT after a 2 h freezing treatment at −20 °C followed by a 3-day recovery at 23 °C (Figure 4A–D). Several important physiological markers including malondialdehyde (MDA) level, proline content, and electrolyte leakage (EL), which have been widely considered to be indicators of tolerance to abiotic stresses, were subsequently measured in transgenic *Arabidopsis* plants and WT. EL could reflect the damage to the cell membrane, and maintaining the integrity of the cell membrane is critical for cold tolerance. Consistent with the phenotype following cold treatment, significantly higher levels of EL were detected in *HbBIN2*-overexpressing transgenic *Arabidopsis* lines than in the WT (Figure 4E,H), implying an exacerbation of cold-induced membrane injury in *HbBIN2*-overexpressing transgenic lines. As an osmoprotectant and stabilizer, proline has been known to protect cells from abiotic stress damage. When developed under normal growth conditions, both HbBIN2-overexpressing transgenic lines and WT exhibited identical proline content, but following cold stress, the transgenic lines’ proline content was noticeably lower than that of WT (Figure 4F,I). MDA was also assessed as an indicator of oxidative damage. As shown in Figure 4G,J, the MDA accumulation increased more rapidly in *HbBIN2-OE Arabidopsis* plants than in the WT during exposure to cold stress conditions for 24 h and 48 h, indicating a higher degree of membrane lipid peroxidation in *HbBIN2-OE Arabidopsis.* These results indicated that overexpression of *HbBIN2–1* or *HbBIN2–3* decreased the tolerance of *Arabidopsis* to cold stress.

### 2.5. Overexpression of HbBIN2s Accumulates More ROS under Cold Stress

**Figure 5:**
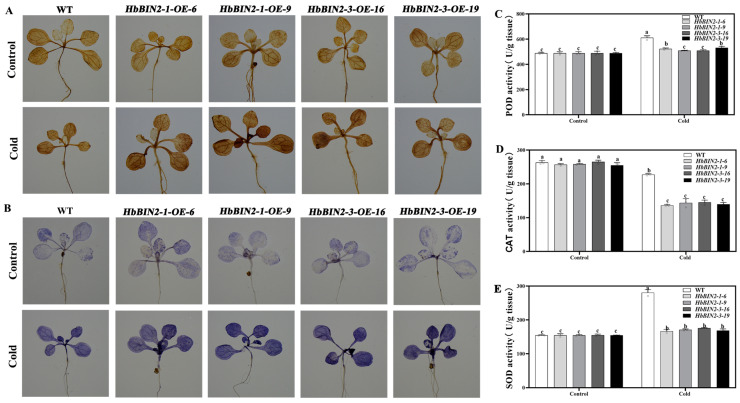
Effects of *HbBIN2* overexpression on regulating the ROS level and the antioxidant system under cold stress conditions in *Arabidopsis.* (**A**,**B**) H_2_O_2_ and O_2_^•−^ in leaves were stained by DAB and NBT before and after cold stress at 4 °C for 24 h. (**C**–**E**) Effects of cold stress on POD activity (**C**), CAT activity (**D**), and SOD (**E**) in the transgenic and WT plant treated with or without cold stress for 24 h. All data are presented by means ± SD of three independent biological replicates. Different letters above the bars indicate significant differences at *p* < 0.05 (Duncan’s range test).

Several stress-related injuries have been linked with ROS-induced damage from oxidative stress. Stress-induced ROS caused the denaturation of lipids, leading to MDA accumulation. Thus, the increase of MDA accumulation in *HbBIN2*-overexpressing transgenic lines might reflect that more ROS are produced in the transgenic plants under low-temperature stress. ROS accumulation in the *HbBIN2-OE* plants and WT was, therefore, measured by diaminobenzidine (DAB) and nitroblue tetrazolium (NBT) staining. As expected, equivalent staining by NBT and DAB was detected in the transgenic lines and WT plants without the low-temperature conditions, whereas after 24 h of cold exposure, transgenic plants showed higher stains than WT plants. (Figure 5A,B), indicating that *HbBIN2-OE* plants accumulated more H_2_O_2_ and O_2_^•−^ than WT plants did. Antioxidant enzymes such as superoxide dismutase (SOD), catalase (CAT), and peroxidase (POD), are known to have a crucial role in scavenging ROS. It was also found that SOD, CAT, and POD functions in *HbBIN2-OE* plants were equivalent to the ones in the WT under normal growth conditions but considerably lower than those in the WT during cold treatment (Figure 5C–E). These results suggested higher levels of damage in the *HbBIN2*-overexpressing transgenic *Arabidopsis* under cold stress compared with the WT plants.

### 2.6. HbBIN2–1 and HbBIN2–3 Negatively Regulate Cold-Responsive Genes Expression under Cold Stress

**Figure 6:**
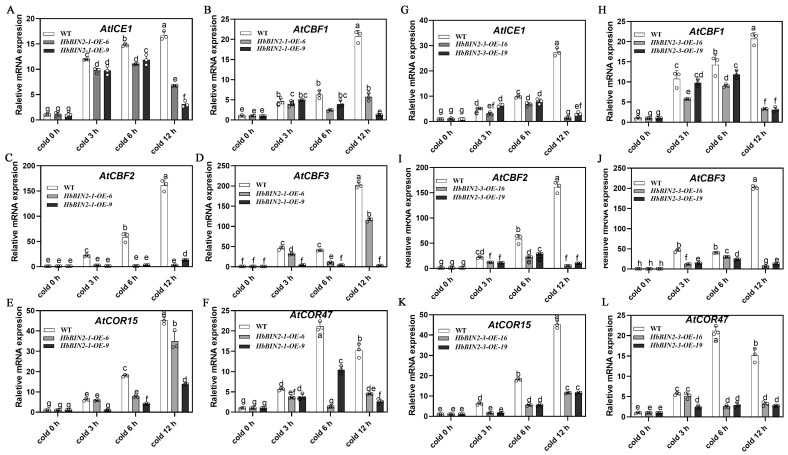
Expression of the cold-regulated genes of the CBF pathway in cold-stressed *HbBIN2* overexpressing *Arabidosis* and WT plants. (**A**–**L**) Expression analysis of *AtICE1*, *AtCBF1*, *AtCBF2*, *AtCBF3*, *AtCOR15,* and *AtCOR47* in 2-week-old WT and *35S::HbBIN2 Arabidopsis* plants treated at 4 °C for the indicated time. Data are presented as means ± SD of three independent biological replicates. Different letters above the bars indicate significant differences at *p* < 0.05 (Duncan’s range test).

The ICE-CBF signaling pathway is vital for plant cold tolerance. To ascertain if the CBF signaling pathway participates in HbBIN2-regulated plant cold stress responses, we examined the transcript levels of the cold-responsive genes expression (*ICE1*, *CBF1*, *CBF2*, *CBF3*, *COR15*, and *COR47*) in the ICE-CBF signaling pathway. As shown in Figure 6, under normal conditions, the base expression levels of cold-responsive genes expression (*ICE1*, *CBF1*, *CBF2*, *CBF3*, *COR15*, and *COR47*) in *35S::HbBIN2* transgenic *Arabidopsis* lines were similar to those in the wild-type plants. However, when exposed to cold stress, the expression levels of all these cold-responsive genes tested were reduced in *HbBIN2*-overexpressing plants compared with those in wild-type. These results indicate that HbBIN2–1 and HbBIN2–3 negatively regulate the expression of cold-responsive genes under cold stress, thereby resulting in reduced cold resistance.

### 2.7. Interactions between HbBIN2s and HbICE1 Repress Transcriptional Activity

**Figure 7:**
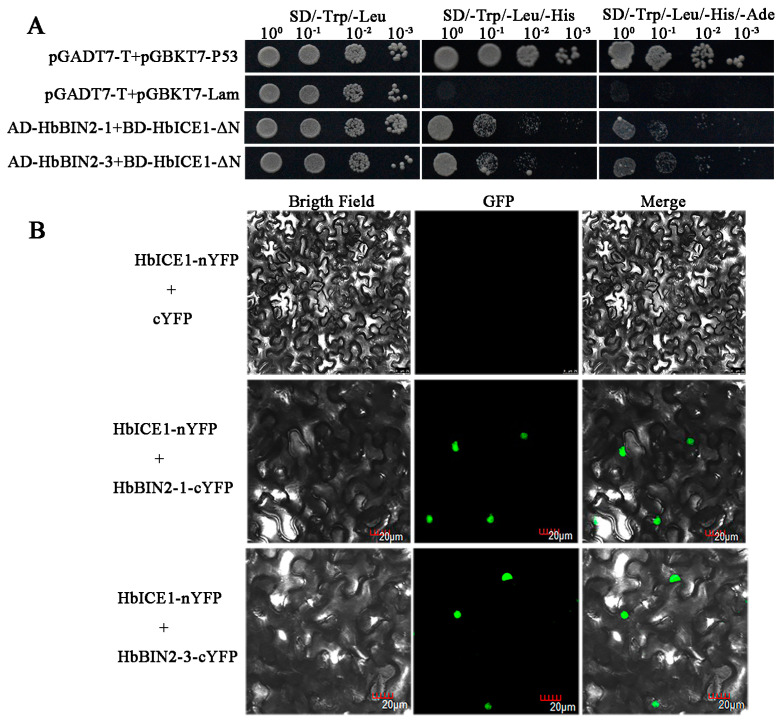
HbBIN2s interact with HbICE1. (**A**) Interaction between HbBIN2 and HbICE1 examined by yeast two-hybrid assay. HbICE1-∆N is truncated HbICE1, which lacks the transactivation region at aa 1–34. The combination of pGADT7-T+ pGADT7-P53 was used as a positive control, and pGADT7-T+ pGADT7-Lam was as a negative control. (**B**) HbBIN2s interact with HbICE1 in *Hevea brasiliensis* protoplasts as indicated by BiFC assay.

To shed light on the molecular mechanism of *HbBIN2*-modulated plant cold resistance, we carried out a yeast two-hybrid screening assay to identify HbBIN2–1-interacting proteins. HbICE1 was screened as one of the candidate interaction proteins of HbBIN2–1. Our previous report showed that HbICE1 is a transcription factor that improves the cold resistance of rubber trees [39]. Thus, we speculated that HbBIN2 might function in the cold stress response by interacting with HbICE1.The truncated HbICE1(HbICE1-∆N, deletion of the transactivation region at aa 1–34) and full-length coding sequence of HbBIN2s were cloned into pGBKT7and pGADT7, respectively, and then transformed into yeast cells to confirm the interaction of HbBIN2s and HbICE1 by point-to-point Y2H assay. Our results show that the yeast cells co-expressed with HbBIN2–1/HbBIN2–3 and truncated HbICE1 were able to grow on SD/-Leu-Trp-His and SD/-Leu-Trp-His-Ade, while negative control (pGADT7-T+ pGADT7-Lam) could not survive on this selection medium, suggesting that HbBIN2–1/HbBIN2–3 interacts with HbICE1in yeast (Figure 7A). The interaction between HbBIN2–1/HbBIN2–3 and HbICE1 was further validated using a bimolecular fluorescence complementation (BiFC) assay. Our confocal microscopy results showed that co-expression of the HbICE1-nYFP and HbBIN2–1-cYFP or HbBIN2–3-cYFP in *N. benthamiana* leaves produced a strong fluorescence signal in the nucleus (Figure 7B). Thus, both in vitro and in vivo studies revealed that HbBIN2–1 and HbBIN2–3 could physically interact with HbICE1.

### 2.8. HbBIN2s Repress the Transcriptional Activity of HbICE1

**Figure 8:**
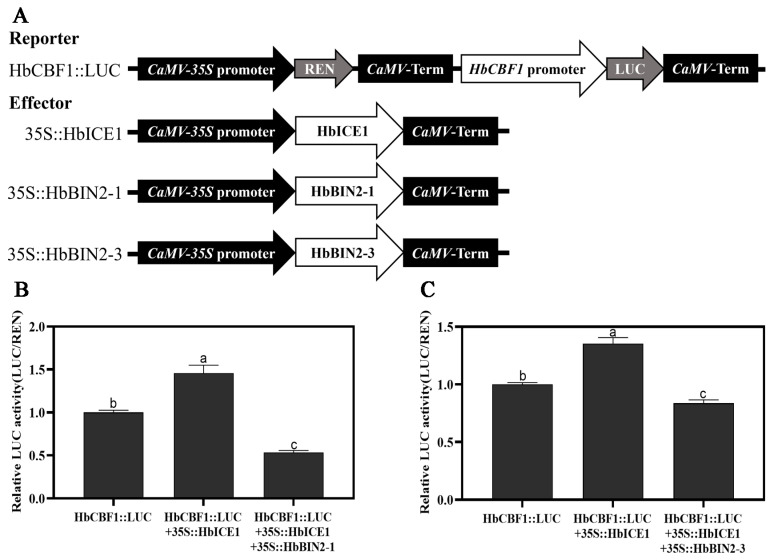
HbBIN2s attenuate HbICE1 transcriptional activity. (**A**) Illustration of effector and reporter constructs used in the transient transactivation assay. (**B**,**C**) Relative LUC activity (LUC/REN) indicating *HbCBF1* expression level measured in tobacco plants harboring indicated plasmids after exposure to 4 °C for 4 h following infiltration for two days. Different letters above the bars indicate significant differences at *p* < 0.05 (Duncan’s range test).

HbICE1 is an important transcription factor that induces *HbCBF* expression by binding to the promoter of *HbCBF* in plant cold stress response. Our above results show that HbBIN2 proteins directly interact with HbICE1, raising a possibility that the physical interactions between HbBIN2 proteins and HbICE1 might affect the transcriptional function of HbICE1. Thus, the reporter plasmid containing the *HbCBF1* promoter-driven firefly luciferase (LUC) and effector plasmids expressing full-length CDS of HbBIN2s or HbICE1 under constitutive 35S promoter were constructed to perform a dual luciferase (LUC) assay in *N. benthamiana* leaves. The LUC/REN ratio showed that HbICE1 considerably increased the promoter activity of *HbCBF1*. However, HbICE1-induced *HbCBF1* expression was drastically inhibited when *HbCBF1::LUC* was expressed together with HbBIN2–1 or HbBIN2–3 and HbICE1 (Figure 8). These results demonstrate that HbBIN2 proteins repress the transcriptional activity of HbICE1.

## 3. Discussion

The tropical tree species known as the rubber tree (*Hevea brasiliensis*) is important economically as the only source of natural rubber used in industry is the latex that is produced by its laticifers. Rubber trees, being a characteristic tropical rainforest tree species, are extremely vulnerable to cold temperatures. Cold temperature lowers natural rubber yield as well as endangers the viability of these plants. Therefore, identifying the potential genes from rubber trees that function in cold response is critical for breeding rubber varieties resistant to cold stress. Previous studies have suggested that the BR signaling pathway regulates cold stress responses [21,41,42]. BIN2 is a major inhibitor of the BR signaling pathway [43,44], Nevertheless, the role and fundamental process of *BIN2* in the cold stress of *Hevea brasiliensis* remain unknown. We explored the involvement of HbBIN2s in cold tolerance in this study.

Glycogen synthetase kinase GSK3, also known as hairy kinase (SK), is a versatile kinase that was originally discovered in rabbits [45]. In plants, BR-INSENSITIVE 2 (BIN2/SK21) is the best-known representative of GSK3 [29]. Our present research revealed that transgenic *Arabidopsis* lines that expressed *HbBIN2* experienced far more severe cold stress damage than wild-type varieties (Figure 4). This indicates that HbBIN2, a repressor of the BR signaling pathway, can be a regulator that negatively impacts cold stress response in plants. In agreement with this, the exogenous application of BR could increase *COR* expression and enhance the cold tolerance of *Arabidopsis* [46]. Our further investigation revealed that *HbBIN2* negatively regulates cold tolerance by interacting with transcription factor HbICE1 and repressing its transcriptional activity, thereby attenuating cold-induced *HbCBF* expression (Figure 8), which is consistent with the previous report that *BIN2* in *Arabidopsis* inhibits the transcript level of *CBF* by BZR1 and CESTA transcription factors [42]. Recent work revealed that in tomatoes, BIN2 reduces tolerance to cold by blocking ABA production [47]. Several studies have shown that there are multifaceted interactions between BIN2 and ABA in *Arabidopis*. For example, protein phosphatase type 2C ABI1 from the ABA pathway inhibits BIN2 kinase activity by dephosphorylation, and BIN2, in turn, phosphorylates SnRK2.2 and SnRK2.3, acting as a positive regulator of the ABA signaling pathway [27,48]. BIN2 also phosphorylates and stabilizes ABI5, an important concentrator of ABA signals [49]. Our previous work showed that exogenous ABA application significantly enhanced *Hevea brasiliensis* cold tolerance [50]. It is very likely that HbBIN2s may also function in *Hevea brasiliensis* cold stress by regulating ABA production or signaling, and the interaction between HbBIN2s and the ABA signaling requires further study. Therefore, HbBIN2 may be employed as a potential gene to generate rubber varieties with improved cold stress tolerance by using RNAi approaches or the CRISPR/Cas9 genome editing system.

Unlike humans, which have only two GSK3 isoforms, plants have evolved multiple GSK3 homologs [29,51]. *Oryza sativa* and *Arabidopsis* have 9 and 10 GSK3-like kinases, respectively [29,52]. BIN2 has two homologs in *Arabidopsis*, BIL1 and BIL2, which function in combination with BIN2 to adversely inhibit the BR signaling pathway [53]. In cotton, six *BIN2* homologous genes have been found [54]. In *Hevea brasiliensis*, four *HbBIN2s* were identified in the present study. Multiple copies of *BIN2* in plants implied that *BIN2* could exert a more diverse role after gene duplications during the long-term evolution of plants. According to this study, all four HbBIN2s in *Hevea brasiliensis* have GSK3-like kinase domains, and the kinase domains and C-terminal sections are largely conserved, whereas the N-terminal areas vary, which is consistent with GSK3-like kinases in other plants. Amino acid sequence alignment revealed that HbBIN2s shared striking sequence similarity with BIN2 in *Arabidopsis* and other plants, suggesting that HbBIN2s we identified are putative BIN2 homologs of *Hevea brasiliensis*.

Our study showed that HbBIN2 could interact with the HbICE1 transcription factor, inhibiting HbICE1 transcript activity. Consistent with this, GSK3s have been previously shown to influence the transcriptional activity or the capacity of transcription factors to bind DNA, such as BZR1, auxin response factor ARF2, and rice growth-regulating factor GRF4 [55,56,57]. Yet, it is still unclear how HbBIN2 affects HbICE1 transcript activity. Earlier studies have reported that BIN2-mediated phosphorylation of the transcription factors is essential for plant development and responses to stress [7,58,59]. In *Arabidopsis*, BIN2-mediated phosphorylate of ICE1 dramatically reduced the binding affinity of ICE1, demonstrating that BIN2 negatively regulates the ICE1 transcript activity partially by phosphorylation [28]. We speculated that HbBIN2-mediated phosphorylate of HbICE1 may affect the transcript activity of HbICE1 in *Hevea brasiliensis* cold stress response, but additional experimental evidence is needed to draw a firm conclusion.

Cold stress always induces osmotic and oxidative stress that results in cell injuries [7]. By acting as an osmoregulatory agent, proline has been shown to protect plants against osmotic stress damage [60]. Therefore, to some extent, proline content in plants reflects stress tolerance, and plants with strong resistance accumulate more proline. In agreement with decreased cold tolerance of *HbBIN2* transgenic lines, *35S::HbBIN2* seedlings exhibited lower proline accumulation than the WT after cold stress treatment, implying that *HbBIN2* overexpression could not effectively adjust osmotic potential and, thus, aggravate cold-induced injuries. Furthermore, abiotic stimuli such as cold stress can cause an overproduction of reactive oxygen species (ROS), leading to stress-induced oxidative damage or even plant cell death [61,62,63]. Moreover, it was observed that cold stress-induced H_2_O_2_ and O_2_^•−^ accumulation in *HbBIN2* transgenic plants was considerably greater than in WT plants following cold treatment (Figure 5A,B), which is consistent with their higher EL and MDA level (Figure 4F–J), supporting the aggravation of cell membrane damage. The results that cold-induced ROS accumulation in WT seedlings could be significantly enhanced by overexpression of *HbBIN2* also imply another function of *HbBIN2* in plant cold stress response by regulating intracellular ROS homeostasis. In line with this, previous reports showed that GSK3 regulated ROS levels by activating Glc-6-phosphate dehydrogenase (G6PD) activity in *Arabidopsis* salt stress resistance [64]. However, how *HbBIN2* regulates ROS balance in plant cold tolerance needs to be further investigated.

## 4. Materials and Methods

### 4.1. Plant Material and Growth Conditions

The *Hevea brasiliensis* clone ReYan 7-33-97 seedlings utilized in this study were provided by the Chinese Academy of Tropic Agricultural Sciences. For expressional analysis of *HbBIN2* genes in different tissues, different tissues (roots, leaves, latex, bark, stems) were obtained for extraction of RNA from three individual plants. Six-month-old *Hevea brasiliensis* seedlings were placed in a plant development chamber at a temperature of 10 °C with 80% humidity for 3, 6, 12, and 24 h to assess the expression of *HbBIN2* genes in response to cold stress, and then leaves were collected for RNA extraction.

*Arabidopsis* seeds were sterilized with 5% bleach for five minutes and washed with sterile water three to four times. Then, they were kept in dark conditions at 4 °C for three days. Finally, they were sown into 1/2 MS medium (with a pH of 5.8) supplemented with 1% sucrose. The seedlings were grown at 23 °C with 16 h of light and 8 h of darkness. Freezing tolerance assays were conducted as per earlier studies with slight alterations [65]. Two-week-old WT *Arabidopsis* seedlings and *HbBIN2-OE* transgenic lines were pretreated at 4 °C for 3 days and then exposed to −20 °C for 2 h. The survival rates were scored after the cold-treated seedlings were transferred to normal conditions for 3 days.

### 4.2. HbBIN2 Gene Identification and Sequencing Analysis in Hevea brasiliensis

To identify *HbBIN2s* from *Hevea brasiliensis*, the complete coding sequence of *AtBIN2* in *Arabidopsis* was used as the query sequence to perform BLAST algorithm-based searches against the *Hevea brasiliensis* genome database. Four *BIN2* genes were identified, which were designated as *HbBIN2–1*, *HbBIN2–2*, *HbBIN2–3*, and *HbBIN2–4*. Multiple sequence alignments of BIN2s protein sequences from *Hevea brasiliensis*, *Arabidopsis thaliana*, cotton, soybean, and rice were conducted by DNAMAN 6.0 software. MEGA 6.0 software was used to generate a phylogenetic tree with the neighbor-joining method.

### 4.3. RNA Extraction and qPCR Analysis

Total RNAs were extracted from *Hevea brasiliensis* and *Arabidopsis* using the Plant RNA Extraction Kit instructions (BioTeke, Beijing, China). The process synthesis of first-strand and qRT-PCR was carried out according to the method outlined in our previous work [39]. The constitutively expressed genes *AtEIF4* and *HbeIF2* were used as internal reference genes for *Arabidopsis* and *Hevea brasiliensis*, respectively. Each of the experiments was repeated at least three times. The primer sequences are displayed in Table S1.

### 4.4. Subcellular Localization of HbBIN2s

*HbBIN2* coding sequences were amplified and inserted into the pBWA(v)BS vector (*35S::GFP*) by homologous recombination to create the recombinant plasmid *35S::GFP-HbBIN2*. After sequence conformation, the recombinant plasmid and *35S::GFP* plasmid (control) were transformed into *Agrobacterium tumefaciens* strain GV3101. After that, *Nicotiana benthamiana* leaves were injected with GV3101 carrying 35S::GFP-HbBIN2 or 35S::GFP plasmid, which were then incubated at room temperature for two to three days. The green fluorescence was observed on the leaves using a Leica TCS S98 confocal laser-scanning microscope. Protoplasts were obtained from *Hevea brasiliensis* leaves and isolated according to the previously reported methodology [66] to study HbBIN2 subcellular location under a confocal laser scanning microscope (Leica TCS SP8), and GFP fluorescence was recorded 14–18 h following transfection.

### 4.5. Generation of Transgenic Arabidopsis

To generate *HbBIN2-OE* transgenic *Arabidopsis* plants, the complete coding sequence of *HbBIN2* was first amplified and then cloned into pBWA(v)BS vector, generating the construct *35S::GFP-HbBIN2*. This construct was subsequently transformed into WT *Arabidopsis* through *Agrobacterium tumefaciens*-mediated transformation and the floral dip method [67]. The positively transformed lines were screened by phosphinothricin (Basta) resistance and then verified by PCR. T4 transgenic plants were utilized to investigate gene function further.

### 4.6. Physiological Measurements and Histochemical Staining

*Arabidopsis* plants were treated at 4 °C for the times specified in the figure legends, and the concentrations of physiological indicators and antioxidant enzyme activity were evaluated. According to our prior findings, electrolyte leakage (EL) and proline buildup were determined [39]. Malondialdehyde (MDA) levels were determined using the thiobarbituric acid (TBA) method, which was previously published [68]. Histochemical staining with diaminobenzidine (DAB) and nitroblue tetrazolium (NBT) was employed to assay H_2_O_2_ or superoxide anion accumulation in situ according to the previous description [69]. Catalase (CAT), peroxidase (POD) activities and superoxide dismutase (SOD) were determined according to the kit manufacturer’s instructions (Nanjing Jiancheng Bioengineering Institute, Nanjing, China).

### 4.7. Yeast Two-Hybrid Assays

The full-length coding sequences of HbBIN2s and truncated HbICE1 (HbICE1∆N, deletion of the transactivation region at aa 1–34) were cloned into pGADT7 and pGBKT7, respectively, to generate recombinant vectors pGAD-HbBIN2s and pGBK-HbICE1∆N. The yeast transformation was carried out using the LiAc technique as described in the MATCHMAKER User Manual (Clontech, San Francisco, CA, USA). The yeast transformants were evaluated for possible interactions on a selective SD medium at 28 °C for 3–4 days.

### 4.8. Bimolecular Fluorescence Complementation Assays

The coding sequences without stop codon of HbBIN2s and HbICE1 were cloned into pSPYCE-35S and pSPYNE-35S vectors, respectively, resulting in 35S::HBIN2-cYFP and 35S::HbICE-nYFP. The recombinant plasmids were transiently injected into *Nicotiana benthamiana* leaves using the combinations shown in the figures. The fluorescence signal was examined with a Leica TCS SP8 confocal microscope after three days of transformation (excitation and emission wavelengths were 480 nm and 550 nm, respectively).

### 4.9. Dual Luciferase Assays

The reporter structure is 35S::REN-pHbCBF1::LUC, which was previously constructed [36]. The full-length CDSs of HbBIN2s and HbICE1 were cloned into pBWA(v)BS and pEGAD vectors to form effector plasmids, and their expression was driven by the CaMV 35S promoter. As previously mentioned, the transient expression was carried out in tobacco leaves [36]. After being cultured for 2–3 days at room temperature and then exposed to 4 °C for 4 h, the activities of the luciferases firefly luciferase (LUC) and Renilla (REN) were measured using a Dual-Luciferase Reporter Gene Assay Kit (Beyotime, Shanghai, China) following the instructions provided by the manufacturer.

### 4.10. Statistical Analysis

All the data, means ± standard deviations (SDs), were analyzed by Student’s *t*-test and Duncan’s range test. Asterisks represent statistically significant differences (* *p* < 0.05, ** *p* < 0.01, and *** *p* < 0.001, Student’s *t*-test). Different letters indicate significant differences in comparison to the control at *p* < 0.05.

## 5. Conclusions

**Figure 9:**
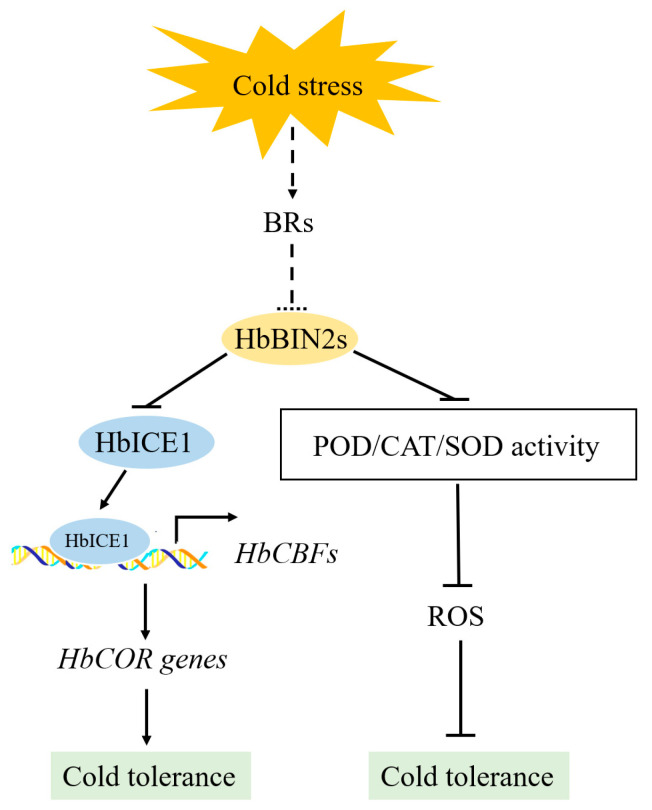
Model of the regulatory function of *HbBIN2s* in response to cold stress. Under cold conditions, HbBIN2s repressors in BRs signaling interact with the HbICE1 transcription factor and inhibit its transcriptional activity. Consequently, they downregulate the expression of its regulons (*CBF1*, *CBF2*, *CBF3*) and CBF’s regulons (*COR15*, and *COR47*) in the ICE-CBF signaling pathway, ultimately diminishing the plant’s tolerance to cold stress. On the other hand, HbBIN2s inhibit the antioxidant system, allowing the cold-induced membrane injury and oxidative damage to be exacerbated by inducing ROS accumulation, thereby decreasing the plant cold tolerance.

In summary, four *HbBIN2s* were isolated from *Hevea brasiliensis* and functionally characterized in this study. The transcript levels of four *HbBIN2s* increased progressively with the duration of cold treatment, suggesting their involvement in the cold stress response of *Hevea brasiliensis*. Furthermore, HbBIN2–1 and HbBIN2–3 were found to be localized in both the nucleus and cytoplasm. Overexpression of *HbBIN2s* in *Arabidopsis* led to decreased cold resistance as evidenced by increased electrolyte leakage, MDA content, ROS accumulation, and reduced survival rates and proline content under cold stress, implying that *35S::HbBIN2s* transgenic plants experienced more severe cold-induced membrane injury and oxidative damage. Moreover, HbBIN2–1 and HbBIN2–3 were shown to interact with the HbICE1 transcription factor, inhibiting its transcriptional activity. These led to the downregulation of the expression of HbICE1′s regulons (*CBF1*, *CBF2*, *CBF3)* and CBF’s regulons (*COR15*, and *COR47*) in the ICE-CBF signaling pathway, reducing plant tolerance to cold stress. Taken together, these findings suggest that either *HbBIN2–1* or *HbBIN2–3* plays a critical role upstream of the ICE-CBF signaling pathway, negatively regulating plant cold resistance through the modulation of HbICE1 transcriptional activity and ROS homeostasis (Figure 9).

## Data Availability

Not applicable.

## Supplementary Materials

The following supporting information can be downloaded at: https://www.mdpi.com/article/10.3390/ijms242115778/s1.
