# Supplementary material for: HbBIN2 Functions in Plant Cold Stress Resistance through Modulation of HbICE1 Transcriptional Activity and ROS Homeostasis in Hevea brasiliensis
Source: Int J Mol Sci. 2023 Oct 30;24(21):15778. doi: 10.3390/ijms242115778 (PMC10649430; doi:10.3390/ijms242115778)
Supplement: Supplementary file 1 [file ijms-24-15778-s001.zip › ijms-2623110-supplementary.pdf]

Supporting Information

Table. S1 List of the Primers Used in this study.

Primer name	Sequence (5' to 3')
**19T-HbBIN2-1 -F**	ggatccATGGCTGATGATAAGG
**19T-HbBIN2-1-R**	ctcgagTCATGTCCCAGCC
**19T-HbBIN2-2-F**	ggatccATGGCTGATGATAAGGAAA
**19T-HbBIN2-2-R**	ctcgagTTATGTCACAGCCAGATGCA
**19T-HbBIN2-3-F**	ggatccATGACATCTGCAGAAGTT
**19T-HbBIN2-3-R**	ctcgagTTAAGAACCAGCTGGATG
**19T-HbBIN2-4-F**	ggatccATGGCTTCTGTGGGTGT
**19T-HbBIN2-4-R**	ctcgagTCATAAGCCAAGAAACGTA
**qRT-HbBIN2-1 -F**	GAAAAGGTGGGGAGGGAGGGA
**qRT-HbBIN2-1-R**	TCAGAGATAGATAGTCACT
**qRT-HbBIN2-2-F**	GCGAGTTGTGGATGGGAGTG
**qRT-HbBIN2-2-R**	ACTGATGGTCTGCTTAGGTTCTC
**qRT-HbBIN2-3-F**	CACTACCATTGGAGGCAAGAATGGG
**qRT-HbBIN2-3-R**	TGCTTCAGGGAAACCACAT
**qRT-HbBIN2-4-F**	TGGTTCTTGAGTATGTCCCTG
**qRT-HbBIN2-4-R**	AGGTTTGATGTCCCGATGA
**qRT-HbeIF2-F**	CGACCTTTGATCCGTTTGCT
**qRT-HbeIF2-R**	CTTCCTACCATTCCGTTGCT
**Genomic-HbBIN2-1 -F**	ATGGCTGATGATAAGG
**Genomic-HbBIN2-1-R**	TCATGTCCCAGCC
**Genomic-HbBIN2-3-F**	ATGACATCTGCAGAAGTT
**Genomic-HbBIN2-3-R**	TTAAGAACCAGCTGGATG
**AD-HbBIN2-1 F**	agagatggatccATGGCTGATGATAAGGGTA
**AD-HbBIN2-1 R**	gacgatctcgagTCATGTCCCAGCCAGGTGC
**AD-HbBIN2-3** F	agagatggatccATGACATCTGCAGAAGTTGAT
**AD-HbBIN2-3 R**	gacgatctcgagTTAAGAACCAGCTGGATGCGG
**PUC-HbBIN2-1 F**	atacggactagtATGGCTGATGATAAGGGTATGT
**PUC-HbBIN2-1 R**	atacggactagtTGTCCCAGCCAGGTGCAA
**PUC-HbBIN2-3 F**	atacggactagtATGACATCTGCAGAAGTTGAT
**PUC-HbBIN2-3 R**	atacggactagtAGAACCAGCTGGATGCGGG
**PSPYCE-HbBIN2-1 F**	atacgc ggatccATGGCTGATGATAAGGGTATGT

**PSPYCE-HbBIN2-1 R**	ataccccccgggTGTCCCAGCCAGGTGCAA
**PSPYCE-HbBIN2-3 F**	atacgcggatccATGACATCTGCAGAAGTTGAT
**PSPYCE-HbBIN2-3 R**	ataccccccgggAGAACCAGCTGGATGCGGG
**qRT-AtCBF1-F**	TGAAGTGAGAGAGCCAAACAAGA
**qRT-AtCBF1-R**	CCGAGTCAGCGAAGTTGAGA
**qRT-AtCBF2-F**	CTCACGACGTCGCCGCCATA
**qRT-AtCBF2-R**	GCTTCAGCCGCCGCCTTTTG
**qRT-AtCBF3-F**	GGCGGAACAGAGCGAAAA
**qRT-AtCBF3-R**	GAAGCGGCAAAAGCATC
**qRT-AtCOR45-F**	GGCGTATGTGGAGGAGAAAG
**qRT-AtCOR45-R**	CCCTACTTTGTGGCATCCTTAG
**qRT-AtCOR45-F**	GGCTGAGGAGTACAAGAACAA
**qRT-AtCOR45-R**	ACAATCCACGATCCGTAACC
**qRT-AtEIF4-F**	GCACAGTTTGATGATGCACGTCAGT
**qRT-AtEIF4-R**	GGTTCTCTTGAAGACCCATGGCA
